# Prognostic role of preoperative D-dimer, fibrinogen and platelet levels in patients with oral squamous cell carcinoma

**DOI:** 10.1186/s12885-021-07841-5

**Published:** 2021-02-05

**Authors:** Yu-jie Liang, Xue-ying Mei, Bin Zeng, Si-en Zhang, Le Yang, Xiao-mei Lao, Gui-qing Liao

**Affiliations:** 1 Department of Oral and Maxillofacial Surgery, Guanghua School of Stomatology, Hospital of Stomatology, Sun Yat-sen University, 56 Lingyuanxi Road, Guangzhou, 510055 Guangdong China; 2 Guangdong Provincial Key Laboratory of Stomatology, No. 74, 2nd Zhongshan Road, Guangzhou, 510080 Guangdong China

**Keywords:** D-dimer, Fibrinogen, Platelets, Oral squamous cell carcinoma, Prognosis

## Abstract

**Background:**

The relationship between cancer and coagulation has been intensively studied in recent years; however, the effects of coagulation factors on oral squamous cell carcinoma (OSCC) have rarely been reported. This study aimed to investigate the relationship between preoperative D-dimer (DD), fibrinogen (FIB), platelets (PLT) and OSCC, as well as the prognostic value of DD, FIB and PLT in OSCC.

**Methods:**

We retrospectively investigated a total of 202 patients with OSCC treated at Guanghua Hospital of Stomatology, Sun Yat-sen University. Baseline demographic and clinicopathological information as well as both preoperative and postoperative DD, FIB and PLT results were collected from each patient, and patients with primary OSCC were followed up for disease progression, death or the end of the study. The correlations between preoperative DD, FIB, PLT and other clinical features, as well as the therapeutic effect and PFS were analysed statistically, and postoperative DD and surgical parameters were also analysed.

**Results:**

Preoperative DD was significantly correlated with T stage, N stage, clinical stage and relapse of OSCC (*P* = 0.000, 0.001, 0.000 and 0.000, respectively). Univariate Cox regression analyses showed that high preoperative DD predicted poor prognosis in patients with OSCC (HR = 2.1, *P* = 0.033), while FIB and PLT showed no prognostic values. Postoperative DD was significantly correlated with preoperative DD and surgical type but not the duration of surgery (*P* = 0.005, 0.001 and 0.244, respectively).

**Conclusion:**

In this study, we suggested that high preoperative DD level may serve as an indicator for synchronous neck dissection in patients with T_1, 2_ OSCC, and the elevated DD level might be the marker of disease progression in patient follow up.

**Supplementary Information:**

The online version contains supplementary material available at 10.1186/s12885-021-07841-5.

## Background

Oral squamous cell carcinoma (OSCC) is one of the most commonly seen malignancies in the head and neck region, and tumour cell proliferation and metastasis occur at high rates even at the very first stage of the primary tumour. Approximately one-half of patients are already in advanced stages of disease when diagnosed [1]. Despite remarkable advances in diagnostic techniques and therapeutic methods, the long-term survival rates of oral squamous cell carcinoma remain poor.

The correlation between cancer and hypercoagulation has been noticed since the nineteenth century, as global haemostasis is more frequently activated in patients with cancer, and much attention has been devoted to this research field. D-Dimer (DD) is a degradation product of the cross-linked fibrin polymer that is sensitive to both coagulation and fibrinolysis activation [2]. Fibrinogen (FIB) and platelets (PLT) can also partly reveal the coagulation state of the patient. Previous studies have reported that pre-treatment plasma DD, FIB and PLT levels can predict prognosis in several types of malignancies, including ovarian [3], breast [4, 5], lung [6, 7], colorectal [8], gastric [9], and pancreatic cancers [10]. However, few studies have reported the use of preoperative DD levels to predict the prognosis of oral cancer. To the best of our knowledge, this is the first study to address the prognostic significance of perioperative DD, FIB and PLT levels and their relationship with clinicopathologic parameters in patients with OSCC.

## Methods

### Patients

This study was carried out at Guanghua Hospital of Stomatology, Sun Yat-sen University. All procedures involving human participants were approved by the hospital Ethics Committee, and written informed consent was obtained. We retrospectively reviewed cases from January 2015 to December 2018. Patients included in the study were pathologically confirmed oral squamous cell carcinoma cases without distant metastasis, and both primary and recurrent cases were analysed. Patients with any of the following criteria were excluded: 1) any other malignancy; 2) previously diagnosed venous thromboembolic diseases; 3) unstable angina; 4) severe infection; 5) history of anticoagulant drug use within 2 weeks before surgery; and 6) patients diagnosed with primary OSCC who had already received previous anti-cancer treatment. Finally, a total of 202 cases were included, and TNM classification was established according to the Union for International Cancer Control (UICC) 2015 guidelines. Preoperative plasma DD, FIB and PLT levels (measured within 1 week before surgery) and postoperative DD, FIB and PLT levels (measured 24 h, 48 h and 96 h after surgery) were collected. Other demographic and clinical information collected included age, sex, tumour location, TNM staging, treatment type, surgical duration, and postoperative adverse effects.

### Treatment and follow-up

Patients were prescribed different treatment schedules according to the 2015 version of the NCCN guidelines for head and neck cancer. Patient follow-up was conducted at three-month intervals for the first 3 years after surgery and then every 6 months until April 2019 until disease progression, death, or loss to follow-up. The study was carried out either by patient follow-up visit or telephone follow-up at each scheduled time.

Progression-free survival (PFS) was chosen as the study endpoint and was defined as the interval from surgery to local or distant relapse and/or metastasis, whichever occurred first. Survival time was considered censored if the patients died, were lost to follow-up, or were progression-free at the end of the study.

### Statistical analysis

Quantitative data are described by the mean (range), and qualitative data are described as counts and percentages. The χ^2^ test was used to evaluate the association between clinicopathologic parameters and preoperative plasma DD, FIB and PLT levels. PFS was estimated by the Kaplan-Meier method, and differences in various prognostic factors were analysed by Cox regression analysis. Univariate analysis was used to identify significant prognostic predictors for PFS, and factors with *P* values of < 0.1 were subjected to multivariate analysis for PFS by Cox proportional hazard analysis. *P* values < 0.05 were regarded as statistically significant. All confidence intervals were set as 95% confidence levels. All statistical calculations were conducted by SPSS version 20.0.

## Results

### Patient characteristics

We collected a total of 202 cases in this retrospective study, including 148 males and 54 females, with a mean age of 56.3 (median 56, range 25–89) years old. Of the 202 patients, 155 had primary OSCC in our department, and the remaining 47 had recurrent lesions after surgical treatment in other medical institutions. The mean follow-up period was 13.62 (median 10, range 1 to 56) months. The clinical parameters of all 202 patients are shown in Table 1, with all the details in the Supplementary file.

**Table 1:** Demographic and clinical characteristics of the patients

Clinical characteristic	Category	N(%)
Sex	Male	148 (73.3)
	Female	54 (26.7)
Age(years)	<60	130 (64.4)
	≥61	72 (35.6)
Tumor site	Tongue	93 (46.0)
	Buccal mucosa	33 (16.3)
	Gingiva	40 (19.8)
	Floor of mouth	18 (8.9)
	Palate	8 (4.0)
	Lip	4 (2.0)
	Lymph node in the neck	6 (3.0)
T stage	T1(primary)	29 (14.36)
	T2(primary)	53 (26.24)
	T3(primary)	26 (12.87)
	T4(primary)	47 (23.27)
	Relapse & metastasis	47 (23.27)
N stage	N0(primary+relapse)	95 + 28 (60.89)
	N+(primary+relapse)	60 + 19 (39.11)
Clinical stage	I	25 (12.38)
	II	40 (19.80)
	III	25 (12.38)
	IV	65 (32.18)
	Relapse	47 (23.27)
Primary or relapse	Primary	155 (76.73)
	Relapse	47 (23.27)
Preoperative D-dimer (μg/L)	Median	358.47
	First and third quality	184.36, 581.74
Preoperative FIB (g/L)	Median	3.56
	First and third quality	2.88, 4.08
Preoperative PLT (10^9/L)	Median	246.5
	First and third quality	210.00, 305.00

### Relationship between preoperative plasma DD, FIB, and PLT levels and clinicopathologic parameters

Of the 202 patients, preoperative FIB and PLT levels were closely related to each other (*r* = 0.376, *P* = 0.000); however, the preoperative DD level and preoperative FIB and PLT levels were not significantly related (*P* = 0.053 and 0.636, respectively).

Preoperative plasma DD, FIB, and PLT levels and clinicopathologic parameters, including age, sex, tumour location, TNM staging, surgical treatment and duration, are summarized in Table 2. The mean preoperative DD level was 499.45 μg/L, and referring to the manufacturer’s recommendation, a plasma DD level of 500 μg/L was set as the cut-off value for normal and high DD values. The mean preoperative FIB and PLT levels were 3.33 g/L and 259.5*10^9/L, respectively, and were set as cut-off values for low and high FIB and PLT values, respectively. In this study, we found that the preoperative DD level in different sex or age groups of patients was not statistically different (*P* = 0.187 and 0.062, respectively); however, in different tumour sites and in different T stage, N stage, and clinical stage patients, the preoperative DD level was significantly different (*P* = 0.040, 0.000, 0.001 and 0000, respectively). However, with regard to preoperative FIB and PLT levels, we found different results: the preoperative FIB level was different based on sex and N stage category only (*P* = 0.025 and 0.002, respectively), and the preoperative PLT level was different based on tumour site and clinical stage category only (*P* = 0.048 and 0.040, respectively).

**Table 2:** Correlation between plasma DD, FIB, PLT levels and patient/tumor characteristics in OSCC cases

Variables	Preoperative DD (μg/L)			Preoperative FIB (g/L)			Preoperative PLT (10^9/L)		
	<500	≥500	*P*	<3.33	≥3.33	*P*	<259.5	≥259.5	*P*
Sex			0.187			0.025			0.114
Male	105	43		51	97		78	63	
Female	34	20		28	26		40	21	
Age			0.062			0.070			0.415
<60	92	33		55	70		70	53	
≥ 60	47	30		24	53		47	32	
Tumor site			0.040			0.387			0.048
Tongue	74	19		42	51		58	28	
Buccal mucosa	18	15		12	21		18	11	
Gingiva	23	17		13	27		22	25	
Floor of mouth	14	4		8	10		11	9	
Palate	4	4		1	7		1	7	
Lip	2	2		2	2		1	1	
Neck (LN)	4	2		1	5		6	4	
T stage			0.000			0.129			0.194
T1(primary)	26	3		14	15		15	9	
T2(primary)	39	14		21	32		36	16	
T3(primary)	23	3		11	15		14	7	
T4(primary)	30	17		22	25		30	28	
Relapse	21	26		11	36		23	24	
N stage			0.001			0.002			0.167
N0(Primary)	75	20		34	61		56	28	
N+(Primary)	43	17		34	26		39	32	
N0(Relapse)	12	16		9	19		15	13	
N+(Relapse)	9	10		2	17		8	11	
Clinical stage			0.000			0.094			0.040
I	22	3		11	14		14	3	
II	30	10		15	25		27	10	
III	21	4		10	15		13	12	
IV	45	20		32	33		41	35	
Relapse	21	26		11	36		23	24	
Primary/Relapse			0.000			0.012			0.113
Primary	118	37		68	87		96	59	
Relapse	21	26		11	36		23	24	

**Fig. 1:**
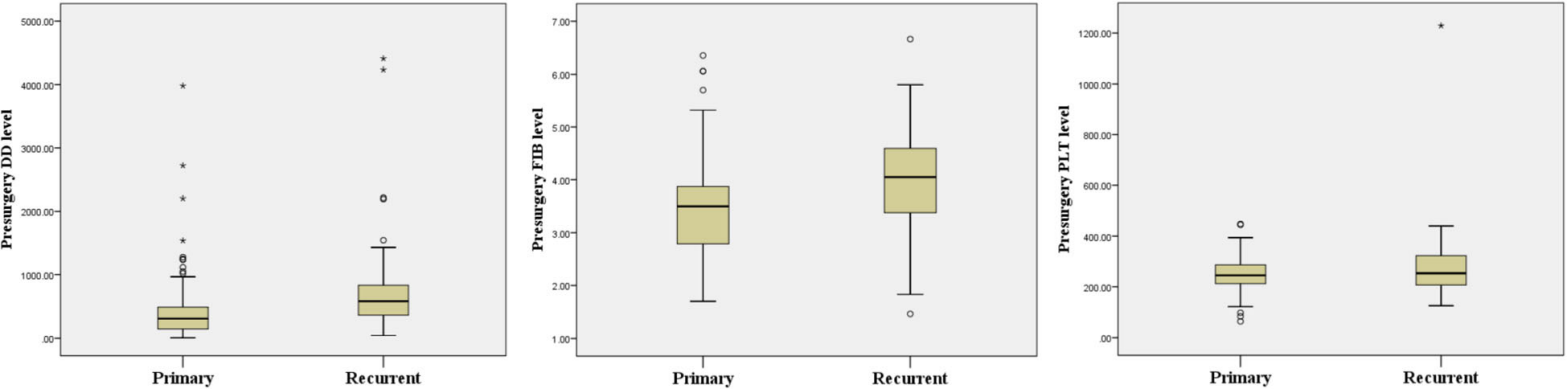
The difference of DD, FIB and PLT levels between primary and recurrent OSCC. *P* = 0.018, 0.038 and 0.062, respectively

The means of DD, FIB and PLT in patients with primary oral cancer were 424.96 μg/L, 3.49 g/L and 249.11*10^9/L, respectively, and those in patients with recurrent tumours were 752.07 μg/L, 3.82 g/L and 283.72*10^9/L, respectively. The difference in DD and FIB levels between primary and recurrent cancer was statistically significant (*P* = 0.018 and 0.038, respectively) (Fig. 1).

### Relationship between postoperative DD change and treatment-related parameters

We retrospectively observed postoperative DD levels in the first 3 days after surgery and in 96 patients with postoperative DD results. The postoperative DD level was elevated in all 96 patients on the first day after surgery and slowly decreased with time. The elevated level was correlated with the preoperative DD level (r = 0.284, *P* = 0.005) as well as the surgical type (r = 0.344, *P* = 0.001) but not the duration of surgery (*P* = 0.244) (Table 3).

**Table 3:** Correlation between postoperative DD change and treatment related characters

	Preoperative DD		Surgery type	Time consuming of surgery		
	r	*P*	r	*P*	r	*P*
Postoperative DD	0.284	0.005	0.344	0.001	0.132	0.244

### Survival analysis of patients with primary OSCC

**Fig. 2:**
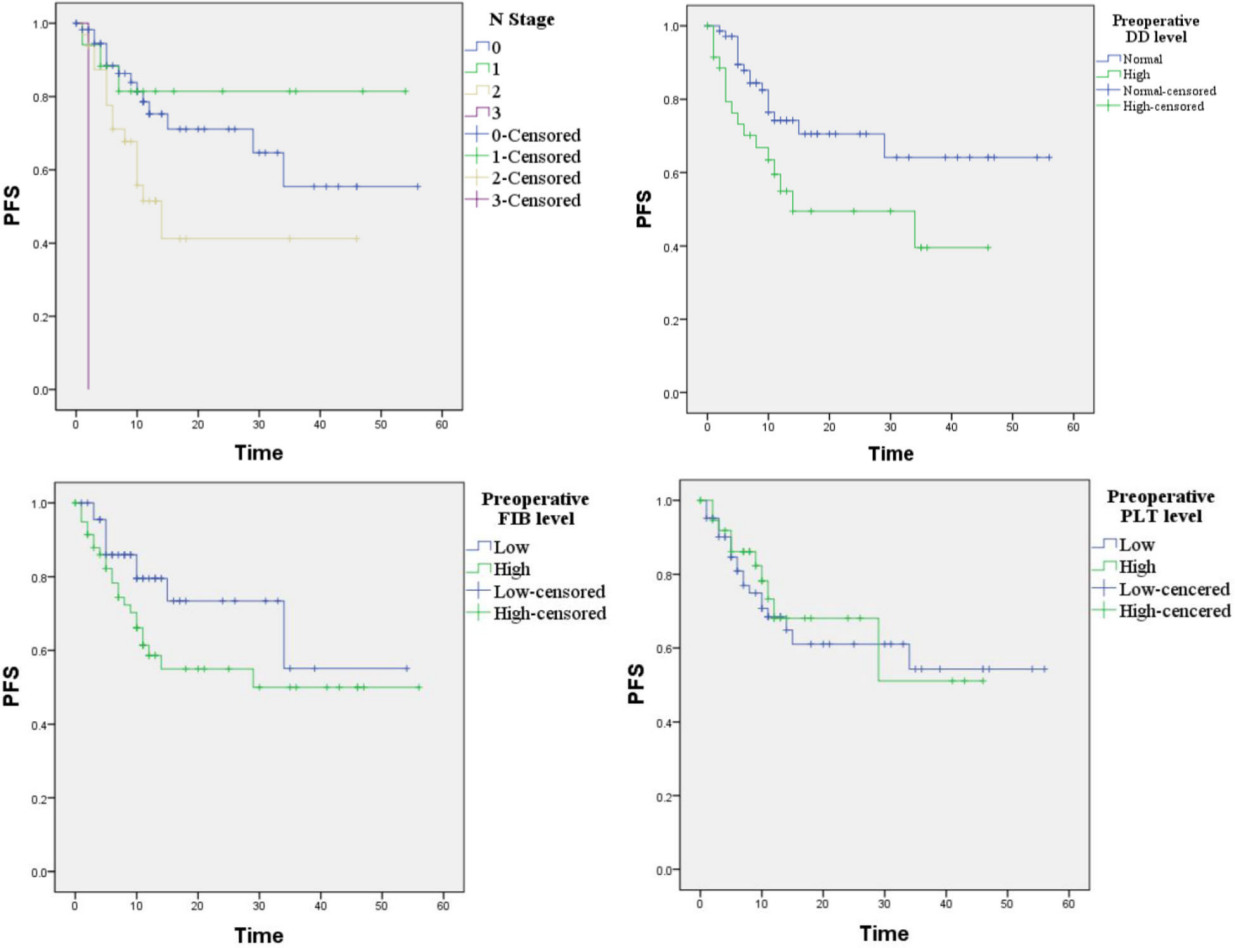
Survival analysis of primary OSCC patients. *P* = 0.003, 0.033, 0.097 and 0.699, respectively

According to the NCCN guidelines and patient desire, 155 patients with primary OSCC were prescribed to different surgical plans including 1) excision of the primary lesion (19.4%); 2) excision of the primary lesion and neck dissection (25%); 3) excision of the primary lesion and vascularized free flap transplantation (0.9%); 4) excision of the primary lesion, neck dissection and vascularized free flap transplantation (51.9%); and 5) unoperated (2.8%). Of the 155 patients with primary oral cancer, recurrence was diagnosed in 33 patients after surgical treatment in our department, rated 21.29%, and 26 of them died during the follow-up time. The time from surgery to disease progression ranged from 1 to 34 months. Univariate analyses revealed that N stage (*P* = 0.003) and preoperative DD level (*P* = 0.033) were predictors of PFS. In multivariate analysis, only N stage was found to be an independent prognostic factor in patients with primary OSCC (*P* = 0.007) (Table 4, Fig. 2).

**Table 4:** Univariate and multivariate analysis for disease-free survival of patients with primary OSCC

Variables	Univariate analysis		Multivariate analysis	
	HR (95% CI)	*P*	HR (95% CI)	*P*
Sex		0.466		
Male	1.00			
Female	0.73 (0.32–1.69)			
Age(years)		0.834		
<60	1.00			
≥ 60	1.08 (0.52–2.23)			
T stage		0.884		
T1 ~ T2	1.00			
T3 ~ T4	1.05 (0.53–2.08)			
N stage		0.003		0.007
N0	1.00		1.00	
N1	0.68 (0.20–2.38)	0.548	0.723 (0.21–2.55)	0.619
N2	2.20 (1.05–4.57)	0.036	2.46 (1.16–5.23)	0.019
N3	33.94 (3.65–315.9)	0.002	19.34 (2.00–186.96)	0.010
Clinical stage		0.499		
I ~ II	1.00			
III ~ IV	1.29 (0.62–2.72)			
Pre. D-dimer		0.033		0.056
<500 μg/L	1.00		1.00	
≥ 500 μg/L	2.10 (1.06–4.16)		1.98 (0.98–3.97)	
Pre. FIB		0.097		0.100
<3.33 g/L	1.00		1.00	
≥ 3.33 g/L	1.88 (0.89–3.95)		1.94 (0.88–4.26)	
Pre. PLT		0.699		
<259.5*10^9/L	1.00			
≥ 259.5*10^9/L	0.86 (0.40–1.84)			
Surgical type		0.732		
1	1.00			
2	1.40 (0.57–3.44)	0.461		
3	1.53 (0.70–3.34)	0.284		
4	0.00 (0.00-)	0.983		
Post.DD change		0.791		
<1000 μg/L	1.00			
≥ 1000 μg/L	1.16 (0.39–3.46)			
Post. PLT change		0.622		
<60*10^9/L	1.00			
≥ 60*10^9/L	1.22 (0.55–2.74)			

* means multiplication

In our study, PFS was 78.7%. Patients with normal preoperative DD (< 500 μg/L) had a significantly better PFS than patients with high preoperative DD (≥500 μg/L) (81.7% vs. 74.2%, *P* = 0.027).

The data that support the findings of this study are available from the corresponding author upon reasonable request.

## Discussion

In this study, we evaluated the relationship between preoperative DD, FIB, and PLT levels and the clinicopathological characteristics of OSCC, as well as the prognostic effect of preoperative DD, FIB and PLT. To the best of our knowledge, this is the first study to address this issue. We found that preoperative DD was significantly different in patients with OSCC with different primary sites or clinical stages, and DD could serve as an independent prognostic factor for patients with OSCC.

However, preoperative FIB and PLT did not show such strong predictive effects. Previous studies have reported that preoperative DD, FIB and PLT have certain prognostic value in several types of cancer [11], including small cell lung cancer [7], hepatocellular carcinoma [6], pancreatic adenocarcinoma [10], gastric cancer [12], and melanoma [13]. PLT was found to contribute to cancer progression through both thrombin-dependent and thrombin-independent mechanisms [14]. FIB is important in blood clotting, fibrinolysis and cellular and matrix interactions [15]. Zheng S et al. found that FIB can enhance PLT adhesion to tumour cells, and PLT in turn can release thrombin and facilitate FIB aggregation [16]. However, unlike most studies of DD, which set the cut-off value to 500 ng/L, there were no consistent cut-off values of FIB and PLT in most of the studies. Hou C et al. [7] set the cut-off values of FIB and PLT as 2.75 g/L and 215*10^9/L, respectively, and they found a marginally significant relationship between elevated PLT and unfavourable PFS (*P* = 0.05) and no prognostic role for FIB. Liu Z and Liu P [6, 10] used 4.0 g/L as the cut-off value for FIB and 300*10^9/L for PLT, and both were significantly correlated with overall survival (*P* < 0.001 and *P* = 0.010, respectively). In a study by Holzinger et al. [17], 447 mg/dL was set as the cut-off value for FIB in patients with oral and oropharyngeal cancer, and elevated FIB was associated with poor overall survival (*P* = 0.005) and recurrence-free survival (*P* = 0.008). In our study, the cut-off values of FIB and PLT were set as the mean value (3.33 g/L and 259.5*10^9/L, respectively). We did find that the FIB level was significantly different between primary and recurrent OSCC, and yet in the survival analysis for primary OSCC, no significant predictive value for PFS was found. We supposed that the inconsistent result between our study and previous studies might be tumour type specific or can partly be attributed to the different sensitivities and different cut-off values of FIB and PLT in different articles.

Cervical lymph node metastasis is the main prognostic factor for patients with OSCC, and neck dissection is an important and effective treatment. However, the complications of neck dissection are severe and significantly lower patients’ quality of life. For cT_1, 2_ N_0_ OSCC patients, even in the NCCN guidelines, there are no definite instructions about performing neck dissection. In this study, the preoperative DD level was found to be significantly correlated with N stage and could serve as an independent prognostic factor for patients with OSCC. Thus, we suggest that a high preoperative DD level may serve as an indicator for synchronous neck dissection in patients with T_1,2_ OSCC.

It is well known that surgical trauma is associated with coagulation, and Friedrich found that DD increased at the end of surgery and remained increased 24 h after surgery [18]. To further reveal the trauma caused by surgical treatment in patients with OSCC, we evaluated the postoperative DD levels and found that postoperative DD was markedly elevated within 24 h and then decreased slowly with time if there were no adverse effects, such as local or general infection or venous thrombosis. Moreover, the rising DD significantly correlated with surgical type but not the duration of surgery. In particular, patients who underwent the most extensive surgery had the highest elevation of postoperative DD level, suggesting that surgical trauma is correlated with the extent of surgery but not the duration of surgery. A relationship between elevated DD and local or general infection and deep vein thrombosis (DVT) was not found in our study, as limited cases developed infection (6/202) and DVT (1/202) after surgery. We also investigated the postoperative DD level with PFS and found that it did not predict PFS.

## Conclusions

DD is an easily measured and reproducible molecular marker. Plasma DD can be routinely measured prior to operations in various hospitals. In this study, we found that high preoperative DD level was significantly correlated with the more advance T and N stage, relapse cases as well as poor PFS in patients with OSCC. We suggest that a high preoperative DD level may serve as an indicator for synchronous neck dissection in patients with T_1, 2_ OSCC and that continuous DD monitoring can be used in OSCC patient follow-up. Certainly, prospective studies and further studies with larger sample sizes are needed to confirm these findings. In addition, postoperative DD can partly reflect surgical trauma and postoperative adverse effects such as infection or venous thrombosis. Therefore, we suggest that preoperative and postoperative DD can be obtained as part of routine care for patients with OSCC.

## Supplementary Information

**Additional file 1.**

## Data Availability

The dataset supporting the conclusions of this article is included within the article and its additional file.

## Abbreviations

DD: D-Dimer
FIB: Fibrinogen
PLT: Platelets
OSCC: Oral squamous cell carcinoma
PFS: Progression-free survival
UICC: Union for International Cancer Control
NCCN: National Comprehensive Cancer Network
